# Serum Metabolomics Profiling to Identify Biomarkers for Unstable Angina

**DOI:** 10.1155/2017/7657306

**Published:** 2017-05-24

**Authors:** Wei Yao, Yuxia Gao, Zheng Wan

**Affiliations:** Department of Cardiology, Tianjin Medical University General Hospital, Tianjin Medical University, No. 154, Anshan Road, Heping District, Tianjin 300052, China

## Abstract

Although statistical evidence is clear regarding the dangerousness of unstable angina (UA), a form of coronary heart disease (CHD) characterised by high mortality and morbidity globally, it is important to recognise that diagnostic precision for the condition is unfavourable. In the present research, to gain insight into candidate biomarkers, the author draws on ^1^H NMR-based serum metabolic profiling to analyze the unstable angina pectoris (UAP) metabolic signatures; this constitutes an effective way to produce medical diagnosis. 101 unstable angina pectoris patients and 132 healthy controls were enrolled and 22 serum samples from each group were analyzed. Effective separation was noted regarding the UAP and control groups, and, for the former group considered in relation to their counterpart, the serum concentrations of Lac, m-I, lipid, VLDL, 3-HB, and LDL were higher whereas the concentrations of Thr, Cr, Cho, PC/GPC, Glu, Gln, Lys, HDL, Ile, Leu, and Val were lower. The conclusion drawn in view of the results is that the plasma metabolomics examined by ^1^H NMR displayed promise for biomarker identification for UA. In addition to this, the analysis illuminated the metabolic processes of UA.

## 1. Introduction

Unstable angina pectoris (UAP) is a frequently encountered complication of coronary heart disease (CHD), and approximately one-third of the population in developed nations experiences the condition prior to turning 70 years old. It results in the hospitalisation of over one million patients each year, and it is a leading factor that contributes to patient deaths [1, 2]. A range of clinical presentations are associated with the condition, and they result from a blockage in the coronary flow. Such blockages can occur as a consequence of various pathophysiological mechanisms, primary among which are intracoronary atheromatous plaque rupture, platelet aggregation, and thrombus formation [3]. At present, the diagnosis of UA takes place with reference to angina symptoms and electrocardiogram modifications [4]. Nevertheless, it is important to recognise the limitations of this approach, which mainly relate to the lack of objectivity regarding symptoms and the nature of the variations in ECG. Despite the fact that coronary angiography is characterised by diagnostic reliability and accuracy for UA, it can only be carried out invasively, thereby meaning that certain individuals are not willing to undergo it. Crucial considerations when diagnosing UAP in clinical practice include patient symptoms, which manifest in the form of high cholesterol, triglyceride-rich lipoprotein particles (primarily VLDL and LDL), and lower levels of cholesterol in HDL particles [5]. However, it is important to note that higher levels are not common to every UA patient and, moreover, they can arise for patients who have different forms of CHD [6].

Research has demonstrated that the pathogenesis of numerous health conditions is linked to metabolite inconsistencies in body tissues and fluids [7]. Metabolomics is useful because it facilitates the quantitative assessment of small molecule metabolites within an organism, and it is possible to employ the method for the purpose of evaluating the way in which the concentration of certain metabolites varies in relation to pathophysiological stimuli [8]. The process has been employed to diagnose CHD with H-nuclear magnetic resonance spectroscopy (NMR), and it facilitates the differential evaluation of the levels of numerous endogenous and exogenous molecules; for this reason, the literature reports that it significantly influences the examination of physiological status, condition diagnosis, biomarker identification, and the detection of the pathways affected by disease or treatment [9]. One of the key quantitative and nondestructive techniques drawn on in clinical settings is high-resolution NMR spectroscopy, which is viewed as advantageous owing to its robustness and reliability, along with the fact that it can be reproduced and repeated [10, 11]. In recent years, this technique has yielded favourable results in biomarker discovery for cardiovascular conditions, including myocardial ischemia [12], heart failure [13], and hypertension [14].

Owing to the way in which it can be obtained straightforwardly from patients in all age groups, serum constitutes an ideal biological fluid for medical examinations. In the present research, ^1^H NMR is applied to serum samples gathered from UAP patients and healthy participants who have previously received diagnosis and confirmation by coronary angiography. Metabolite profile variance is recorded in relation to the serum of each group in view of physiological and pathological differences, and it is important to recognise that advanced characterisation and authentication using a significant sample size could facilitate their establishment as clinically useful biomarkers. 

## 2. Materials and Methods

### 2.1. Plasma Collection

A completely randomized design was used in this research, and the sample size was calculated according to the design. The research received approval from the Ethics Committee of the Tianjin Medical University General Hospital and Tianjin Medical University, and written consent was gathered from every participant. From January 2015 to June 2015, 101 individuals who received coronary angiography for UA diagnosis and diagnosed as UAP at the Department of Cardiology, Tianjin Medical University General Hospital were registered for this study. The inclusion criteria were based on UA diagnosis and selective coronary angiography. Angina symptoms were new in onset, getting progressively worse, or arising with minimal activity, unaccompanied by ECG changes of ST elevation, and angiographically documented organic stenosis *Z* > 75% in a minimum of one major coronary artery. The exclusion criteria include (1) participants had a history of myocardial infarction, coronary revascularisation, heart failure, liver/renal disease, inflammatory conditions, or metabolic disease; (2) participants who were not willing to sign the written consent form were removed; (3) the cases were removed where clinical information was lacking or missing and therefore statistical analysis could not be performed. A 132-person control group, constituted of individuals in full health, was constructed by drawing on voluntary support at the medical examination center at Tianjin Medical University General Hospital.  Table 1 overviews the demographic data pertaining to each of the sample groups.

Before 24 hours had elapsed after being admitted, each patient was set into an overnight fasting state and, the following morning, a 5-mL sample of peripheral venous blood was obtained. Centrifuging was employed at 3000 rpm for a period of 10 minutes at 4°C, and the serum was subject to storage at 80°C while awaiting analysis. Additionally, following the patients' enrolment onto the study, information was gathered regarding demographic data, medical history, personal history, and signs, and data from four conventional diagnostic methods was logged. Notably, the collation of patient histories and the data from diagnostic methods was informed by pertinent specialists.

### 2.2. Sample Preparation and ^1^H NMR Spectroscopic Analysis of Serum

The ^1^H NMR analysis of serum samples took place in the manner as already accounted for [15], and serum samples storage at −80°C took place before 3 hours had elapsed following collection to maintain the samples for urinalysis. 22 samples from UAP group and 22 samples from the control group were randomly selected. Thawing of the samples took place a single time in the context of a biosafety fume hood, and preparation took place by combining 550 *µ*l of serum with 55 *µ*l of 1.5 mol/l deuterated phosphate buffer (NaH_2_PO_4_ and K_2_HPO_4_, including 0.1% TSP (sodium 3-(trimethylsilyl)propionate-2,2,3,3-d4), pH 7.47). Where the serum was not sufficient, D_2_O up to 550 *µ*l was added. In turn, the serum-buffer combination was set to rest for 5 minutes at room temperature, and this was followed by centrifuging at 10,000 rpm at 4°C for a period of 10 minutes, the purpose of which was to remove floating debris. The next step was to transfer the supernatant (550 *µ*l) into a 5 mm NMR tube, and TSP was used as a chemical shift reference (*δ*  0.0), and D_2_O provided a lock signal.

Every NMR spectra were subject to measurement at a ^1^H frequency of 600.11 MHz, and this was carried out by employing a Bruker Avance AVIII 600 spectrometer at 298 K (Bruker Biospin, Rheinstetten, Germany). A conventional one-dimensional (1D) NMR spectrum was used to facilitate water presaturation, and this served as a standard representation of the overall metabolite composition. To weaken signals from macromolecules by the CPMG (Carr-Purcell-Meiboom-Gill) pulse sequence, the researcher employed an interpulse delay of 3 *µ*s, a mixing time of 100 ms, and irradiation of the water resonance. A BPP-LED (bipolar-pair longitudinal eddy) current pulse sequence was employed for the purpose of detecting large macromolecule signals, and a two-dimensional ^1^H-^1^H COSY (correlation spectroscopy) and TOCSY (total correlation spectroscopy) were also performed for selected plasma samples to facilitate resonance assignment.

Before Fourier transformation, an exponential window function of 1.0 Hz was employed to multiply free induction decay (FID), and these were subject to correction for phase and baseline distortions. This was carried out by utilising TopSpin 2.0 (Bruker). Chemical shifts were referenced to the peak of the anomeric proton of *α*-glucose at *δ* 5.23, and NMR spectra (*δ* 0.5–8.5) were subject to binning with regions 0.002 ppm wide; in turn, automatic integration took place by employing the AMIX package (v.3.8.3, Bruker Biospin, Germany). For the purpose of bypassing the impacts associated with imperfect water suppression, the *δ* 4.55–5.13 region was extracted, thereby meaning that the spectra over the ranges *δ* 0.5–4.55 and *δ* 5.13–8.5 were chosen and limited to 3663 regions, each being 0.002 ppm wide. Normalisation took place for every internal region to the total of every integral region for each spectrum before pattern recognition analysis.

Principal component analysis (PCA) was initially employed for detecting the CPMG spectra from each serum sample in order to derive an outline of data distribution and similarities between the samples, for instance, in terms of clustering and outliers. This was facilitated by drawing on the Simca-P 11.0 software (Umetrics, Sweden). Following this, the partial least-squares discriminant analysis (PLS-DA) was utilised with unit variance scaling to facilitate a more in-depth analysis of the NMR spectral data. A tenfold cross-validation method was used to get* Q*^2^ and* R*^2^, as these two values represent predictive ability and the explained variance of the model, respectively. In order to further confirm the validation of the PLS-DA model quality, permutation tests including a randomly permuting class membership and running 200 iterations were carried out. Then, the OPLS-DA was used to maximize covariance between the measured data and the response variable.

### 2.3. Statistical Analysis

The SAS software package (V 9.2, Cary, NC, USA) was employed to conduct statistical tests, and a Proc Mixed analysis of variance-covariance was applied to the data before conducting Tukey's multiple comparisons test. The information is presented in the form of the mean ± standard error of the means, and a* P* value lower than 0.05 is considered as statistically significant.

## 3. Results

**Figure 1:**
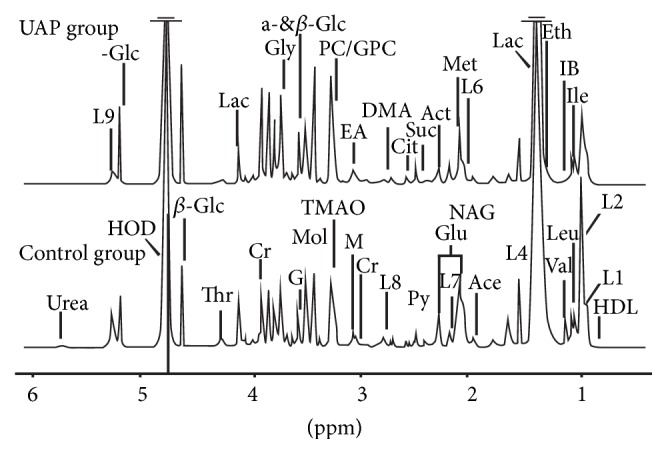
Representative spectra of ^1^H NMR from UAP and control groups. The different metabolites are as follows: 1-MH, 1-methylhistidine; Ace, acetic acid; Act, acetone; Cit, citric acid; Cr, creatinine; DMA, dimethylamine; EA, ethanol amine; Eth, ethanol; For, formic acid; Glc, glucose; Glu, glutamic acid; Gly, glycine; G, glycerinum; GPC, glycerophosphoryl choline; HDL, high-density lipoprotein; HX, hypoxanthine; IB, isobutyrate; Ile, isoleucine; L1, LDL, CH_3_-(CH_2_)_*n*_-; L2, VLDL, CH_3_-(CH_2_)_*n*_-; L4, VLDL, CH_3_-(CH_2_)_n_-; L5, VLDL, -CH_2_-CH_2_-C=O; L6, Lipid, -CH_2_-CH=CH-; L7, Lipid, -CH_2_-C=O; L8, Lipid, =CH-CH_2_-CH=; L9, Lipid, -CH=CH-; Lac, lactic acid; Leu, leucine; Lys, lysine; M, malonic acid; Met, methionine; Mol, methyl alcohol; NAG, n-acetyl-glycoprotein; PC, phosphocholine; Phe, phenylalanine; Py, pyruvic acid; Suc, succinic acid; Thr, threonine; TMAO, trimethylamine; Urea, Uric Acid; Val, valine.

Since serum, when considered in relation to any other biological fluid, is characterised by its high level of availability and metabolite richness following a series of biochemical procedures, it is a useful way in which to gain bio-information regarding an organism's metabolism. As noted, the present study has examined 22 UA patients and 22 AS controls, and the spectra of ^1^H NMR measurements for serum samples from each group are displayed in Figure 1. Previously conducted research along with the in-house NMR database was referenced to facilitate resonance assignments [16, 17].

**Figure 2:**
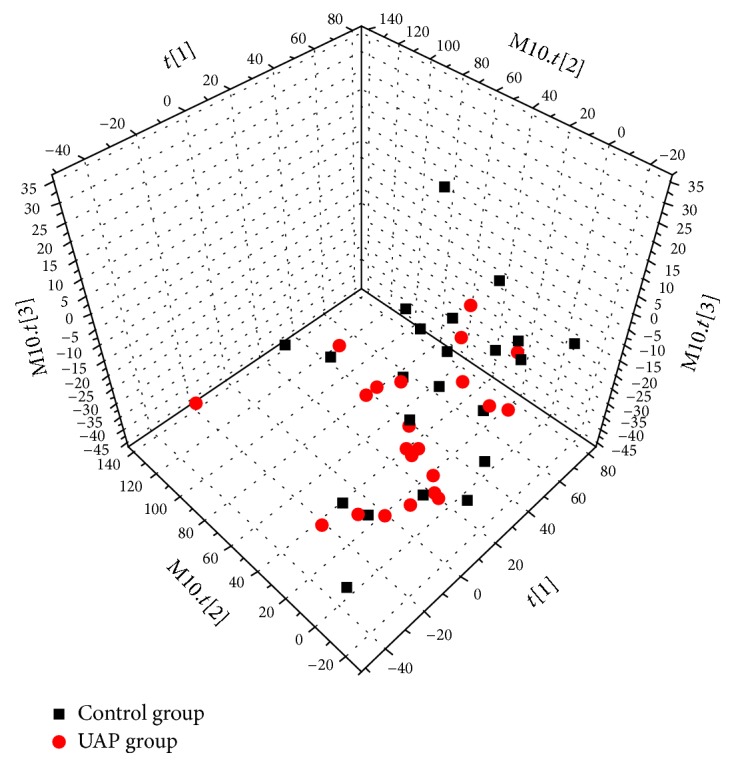
PCA score plot for UAP and control groups; score plots displaying discrimination regarding UAP (red circles) and controls (black squares) (*R*^2^*X* = 0.530 and* Q*^2^ = 0.289).

The first step was to conduct PCA for the purpose of detecting group separation depending on NMR signal variability (see Figure 2; *R*^2^*X* = 53.0%, *Q*^2^ = 28.9%). Figure 2 illustrates an effective separation trend regarding each sample, but a degree of overlying results have been detected; consequently, no significant variance can be observed between the UAP and control groups in terms of the PCA plot score.

**Figure 3:**
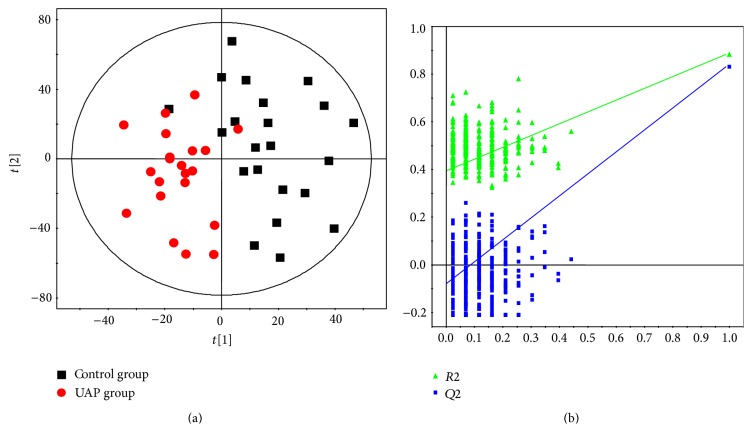
Score plot of PLS-DA (a) for UAP and control groups, score plots indicates the separation degree between UAP (red circles) and controls (black squares) (*R*^2^*X* = 0.550, *R*^2^*Y* = 0.884, and* Q*^2^ = 0.824), and validation of PLS-DA (b). A permutation test is conducted with 200 randomly initiated permutations in a PLS-DA model showing* R*^2^ (green triangles) and* Q*^2^ (blue boxes) values from the permuted analysis (left-bottom corner), and these are far lower than the associated initial values (right-top corner).

PLS-DA is useful in maximizing variance between UAP and control groups and facilitating the screening of metabolites, and this is carried out by eliminating systematic variations which bear no relation to pathological status. PLS-DA model (Figure 3(a)) showed the score plot of UAP and control groups were rendered clearly distinct with *R*^2^*X* = 0.550, *R*^2^*Y* = 0.884, and* Q*^2^ = 0.824. The parameters used to describe the PLS-DA model were considerably heightened, thereby indicating the robustness of the model. For the purpose of validating the model's performance, the author conducted a 200-iteration permutation test.  Figure 3(b) illustrates that the validation plot of the initial PLS-DA model is neither random nor overfitting, and this can be seen with reference to the fact that the permutated* Q*^2^ and* R*^2^ values are far less than the associated initial values.

**Figure 4:**
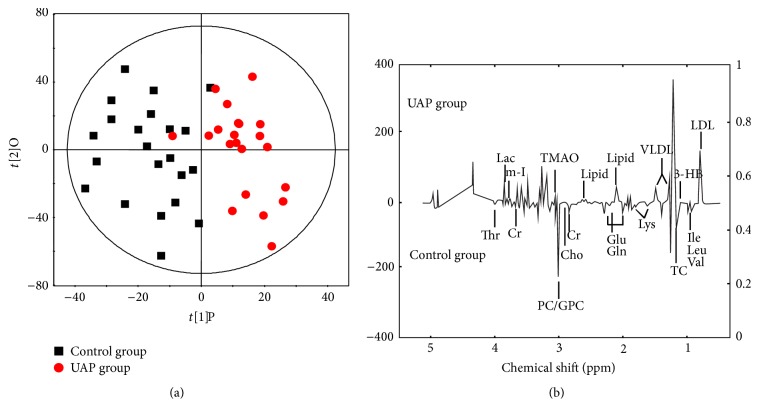
Score plot of OPLS-DA model (a) for UAP and control groups, score plots showing the model's separation regarding UAP (red circles) and healthy controls (black squares), and OPLS-DA corresponding correlation coefficient loading plots (b) of key metabolites.

For the purpose of eliminating the impact of individual variance and, furthermore, to illuminate the modified metabolites governing the separation regarding each group, the OPLS-DA model was formulated. The cross-validation parameters* Q*^2^*Y* were employed to generate a description of the model's quality, and this also served to provide insight into the degree to which the model was predictable; in combination with this,* R*^2^*Y* was used to represent the total explained variation. Regarding score plot of OPLS-DA model (*R*^2^*Y* = 0.894 and *Q*^2^*Y* = 0.877), notable biochemical variance regarding the respective sample groups was observed (see Figure 4(a)), and the metabolic variations for UAP group were summarized in a color-coded coefficient plot (see Figure 4(b)). The metabolites displaying considerable change (*P* < 0.05) were detected on the basis of the absolute cut-off value regarding correlation coefficients. Furthermore, with *r* > 0.423, the serum samples of the UAP group displayed upregulation of Lac, m-I, lipid, VLDL, 3-HB, TMAO, and LDL. Correspondingly, samples displayed downregulation of Thr, Cr, Cho, PC/GPC, Glu, Gln, Lys, TC, Ile, Leu, and Val.  Table 2 presents a summary of the metabolic variation.

## 4. Discussion

It is important to recognise that patients with UA could be provided with pharmaceuticals to facilitate prevention. In order to identify the biomarkers linked to UA and, furthermore, to note the difference between these and the biomarkers stemming from medication, 101 UA patients and a 132-person control group comprised of healthy patients were enrolled and 22 samples from UAP group and 22 samples from control group were analyzed in this research. This methodological approach has also contributed to the development of insight regarding the degree to which biomarkers for UA diagnosis are reliable.

The present research has found that plasma metabolomics with the ^1^H NMR metabolomics technique illuminated the nature of the metabolic differences regarding UAP and control groups. The ensuing examination of the profiles of serum samples from the UAP group had the capacity to differentiate between it and the latter group, and it facilitated the provision of a metabolic fingerprint of the disease. In this way, the promising nature of metabolomic spectrum in evaluating the disease has been emphasised. In addition, the method was employed for the purpose of evaluating the degree to which it is accurate and reliable in UAP diagnosis, and it has demonstrated a more effective performance regarding specificity and sensitivity.

The experimental findings have allowed the author to detect the 18 central metabolites governing the differentiation between UAP and control groups. Regarding these metabolites, the levels of Lac, m-I, lipid, VLDL, 3-HB, TMAO, and LDL were upregulated in UAP group in comparison to the healthy control group, while Thr, Creatine, Cho, PC/GPC, Glu, Gln, Lys, TC, Ile, Leu, and Val were downregulated in relation to the controls. In view of this, it is possible to conclude that phospholipid and amino acid metabolism is disrupted in regard to the UA patients. Park et al. showed increased level of lipid metabolites were associated with a higher risk of myocardial infarction [18]. As phospholipids are a critical feature of every cell membrane, they have the potential to formulate lipid bilayers. In addition to this, phospholipids play a role in varied cell processes, including apoptosis, cell-cell interaction, cell proliferation, and cell differentiation [19]. Recently conducted research also demonstrated that phospholipid metabolism performed a critical function regarding the pathogenesis of metabolic syndrome and hepatic steatosis, and this subsequently resulted in the incremental progression of CVD [20]. Disturbed phospholipid metabolism played an important role in cardiovascular pathophysiology, and this included necrotic core formation, plaque erosion or rupture, and platelet aggregation [21]. In view of this, it is justifiable to conclude that disturbed phospholipid metabolism is critical for the development of UA.

The increased level of serum creatinine is a marker of acute coronary syndrome, in patients with a creatinine level of 1.0 mg/ml have a 10–35% higher motility than the control group [22]. Saygitov et al. also showed that the increased blood urea nitrogen together with serum creatinine level was the independent risk factors of high motility in ACS patients [23]. The majority of creatinine transitions into phosphocreatine and produces ATP by the reversible capacity of the enzyme. It transitions into creatinine in the absence of the enzyme in nonstandard scenarios. This highlights that the lower level of creatinine in the serum of UAP group indicates the inability of UAP group to generate ATP conventionally; consequently, the requirement exists for a greater quantity of creatine to transition into phosphocreatine, thereby producing ATP [24].

The metabolite levels for amino acids in this research, including Thr, Gln, Lys, Ile, Leu, and Val, were considerably reduced for the UA patients when considered in relation to the healthy controls. The energy metabolism of myocardium cells was disturbed in UA patients and these cells have to find alternative substrates to provide energy [25, 26]. Glutamine, a critical resource for gluconeogenesis, is classified as a form of glucogenic amino acid, and glutamine itself is a critical component of the TCA cycle; moreover, it performs a crucial function in numerous metabolic pathways, in particular regarding the maintenance of amino acid homeostasis [27]. Some researches drew on metabolomics profiling for the purpose of comparing cardiac extraction and plasma substrates, and the results indicated that CHD experienced a lower level of glutamate or glutamine [28]. Nishimura et al. showed that Val decreased the damage induced by metabolic disorder and hypoxia in a rabbit model [29]. In the present study, the lower level could potentially result from the amino acid metabolism disorder, where lysine has the potential of reducing the concentrations of blood triglycerides to hinder cardiovascular and cerebrovascular disease.

To conclude, the metabolomics technique has offered a high degree of utility in the context of this initiative to improve the under diagnosis of UAP. The present study's findings indicate 18 possible biomarkers associated with UAP were identified through analysis and serum metabolomics is a highly effective way to detect biomarkers which can facilitate subsequent differentiation regarding UAP patients and controls. In view of this, the technique of metabolomics is valuable in enhancing the under diagnosis of UAP, and it is further notable that the detected metabolite biomarkers, in functioning as predicative factors, have the potential to be used to formulate a classification model; this could be drawn on to facilitate the preliminary diagnoses of UA patients, thereby creating the opportunity to provide personally tailored clinical solutions to patients. At the same time, effective treatment would be facilitated in a viable timeframe, thereby avoiding the further development of the disease to myocardial infarction and cardiac death. Biomarkers of this kind have similar potential to offer further insight into the biological mechanisms of UA, and these can facilitate valuable hints on the basis of which practitioners can heighten treatment standards.

## Figures and Tables

**Table 1:** Demographic and clinical characteristics of unstable angina patients and healthy controls.

	Controls *n* = 132	UAP group *n* = 101	*χ* ^2^/*t*/*U*	*P*
Male	72 (54.55%)	53 (52.48%)	0.099	0.753
Age (years)	61.04 ± 7.38	63.18 ± 9.65	1.265	0.209
Hypertension	71 (53.79%)	60 (59.40%)	0.734	0.392
Diabetes	28 (21.21%)	30 (29.70%)	2.206	0.137
Smoking	40 (30.30%)	48 (47.52%)	7.220	0.007^*∗*^
SBP (mmHg)	136.88 ± 19.62	133.98 ± 17.50	0.792	0.430
DBP (mmHg)	83.10 ± 10.72	79.41 ± 8.53	1.927	0.057
HR (BPM)	69.25 ± 10.95	70.06 ± 10.02	0.391	0.697
BMI (Kg/m^2^)	26.12 ± 3.24	25.14 ± 2.99	0.202	0.117
WC (cm)	97.90 ± 10.11	85.80 ± 8.61	1.119	0.266
LV (mm)	47.22 ± 3.62	47.74 ± 4.82	0.610	0.543
EF (%)	63.98 ± 3.80	62.42 ± 6.72	1.430	0.156
TC (mmol/L)	4.41 ± 1.02	4.32 ± 1.02	0.451	0.653
TG (mmol/L)	1.29 (0.84, 1.59)	1.51 (1.00, 1.84)	2.235	0.025
HDL-C (mmol/L)	1.27 (0.97, 1.52)	1.06 (0.84, 1.27)	2.857	0.004^*∗*^
LDL-C (mmol/L)	2.54 ± 0.89	3.35 ± 0.89	2.026	0.038^*∗*^
GLU (mmol/L)	5.63 (5.00, 6.00)	6.37 (5.10, 6.60)	1.087	0.277
CR (umol/L)	65.65 (56.00, 73.00)	68.98 (58.00, 76.00)	1.047	0.295
UA (mmol/L)	301.63 ± 80.37	309.96 ± 84.25	0.513	0.609

Data are presented as mean ± SD. SBP: systolic blood pressure; DBP: diastolic blood pressure; HR: heart rate; BMI: body mass index; WC: waist circumference; LV: left ventricular end diastolic diameter; EF: left ventricular ejection fraction; TC: total cholesterol; TG: triglycerides; HDL-C: high density lipoprotein cholesterol; LDL-C: low density lipoprotein cholesterol; GLU: blood glucose; CR: creatinine; UA: uric acid. *∗* indicates statistically different between groups.

**Table 2:** OPLS-DA coefficients derived from the CPMG NMR data of metabolites in serum samples obtained from different groups.

Metabolites	1H (ppm) and multiplicity^b^	*r* ^a^ UAP − control
LDL	0.85(br), 1.28(br)	0.751
Glycerophosphocholine	3.23(s)	−0.669
Threonine	4.25(m)	−0.658
Phosphocholine	3.21(s)	−0.626
TC	0.70(br)	−0.624
3-Hydroxybutyrate	1.20(d), 2.31(dd), 2.41(dd), 4.16(m)	0.577
1-Methylhistidine	7.07(s), 7.81(s)	−0.556
Lipid, -CH_2_-C=O	2.24(br)	0.537
Phenylalanine	7.33(d), 7.37(t), 7.42(m)	−0.530
Lipid, =CH-CH_2_-CH=	2.78(br)	0.527
Glutamate	2.08(m), 2.12(m), 2.35(m), 3.78(m)	−0.500
Creatine	3.04(s), 3.93(s)	−0.491
Lysine	1.45(m), 1.71(m), 1.91(m), 3.01(m), 3.76(m)	−0.490
Glutamine	2.14(m), 2.45(m), 3.78(m)	−0.486
Choline	3.20(s)	−0.480
Leucine	0.96(t)	−0.478
Valine	0.99(d), 1.04(d)	−0.473
*myo*-inositol	3.28(t), 3.56(dd), 3.61(m), 4.06(t)	0.468
Isoleucine	0.94(t), 1.01(d)	−0.452
VLDL	0.88(br), 1.30(br), 1.58(br)	0.441
Lactate	1.33(d), 4.11(q)	0.431
TMAO	3.27(s)	0.430

^a^Correlation coefficients: positive and negative signs indicate positive and negative correlation in the concentrations, respectively. The correlation coefficient of |*r* | > 0.423 was used as the cutoff value for the statistical significance based on the discrimination significance at the level of *P* = 0.05 and df (degree of freedom) = 20. ‘‘−” means the correlation coefficient |*r*| is less than 0.423. Multiplicity^b^: s: singlet; d: doublet; t: triplet; q: quartet; dd: doublet of doublets; m: multiplet; br: broad single peak.
